# Evaluation of Antibody Response in Sows after Vaccination with Senecavirus A Vaccine and the Effect of Maternal Antibody Transfer on Antibody Dynamics in Offspring

**DOI:** 10.3390/vaccines9101066

**Published:** 2021-09-24

**Authors:** Fan Yang, Zixiang Zhu, Huanan Liu, Weijun Cao, Wei Zhang, Ting Wei, Min Zheng, Keshan Zhang, Hong Tian, Qiaoying Zeng, Xuepeng Cai, Haixue Zheng

**Affiliations:** 1 College of Veterinary Medicine, Gansu Agricultural University, Lanzhou 730070, China; yangfan02@caas.cn (F.Y.); zengqy@gsau.edu.cn (Q.Z.); 2 State Key Laboratory of Veterinary Etiological Biology, National Foot and Mouth Diseases Reference Laboratory, Key Laboratory of Animal Virology of Ministry of Agriculture, Lanzhou Veterinary Research Institute, Chinese Academy of Agricultural Sciences, Lanzhou 730046, China; zhuzixiang@caas.cn (Z.Z.); liuhuanan2008@163.com (H.L.); caoweijun@caas.cn (W.C.); zhangwei09@caas.cn (W.Z.); tingting830830@163.com (T.W.); zhengmin105251@163.com (M.Z.); zhangkeshan@caas.cn (K.Z.); tianhong@caas.cn (H.T.)

**Keywords:** Senecavirus A, vaccine, maternal antibody, immunization schedule, antibody persistence

## Abstract

Senecavirus A (SVA) is a newly porcine virus that has been detected in many countries since its first detection in pigs in Canada in 2007, and it remains endemic in many countries in Asia and America, which has become a substantial problem for the pig industry. Vaccination is a potentially effective strategy for the prevention and control of SVA infection. Our lab has developed a SVA vaccine candidate previously. In this study, the antibody response to the prepared vaccine in sows and their offspring was evaluated. Vaccination of sows with inactivated SVA vaccines during pregnancy elicited SVA-specific virus-neutralizing antibodies. Vaccination with a high dose of SVA vaccine followed a booster immunization contributed to a long-term duration of the persistence of maternally derived neutralizing antibodies (MDAs) in the milk of the sows (>14 days). In contrast, vaccination with a single low dose of SVA vaccine resulted in a short-term persistence of MDAs in the milk (2–7 days). The MDAs could be efficiently transferred from the sows to their offspring through the colostrum/milk but not the umbilical cord blood. The antibody titers and the duration of the persistence of MDAs in the offspring are highly associated with the antibody levels in the milk from the sows. Vaccination of sows with a booster dose of SVA vaccine resulted in a longer-lasting MDAs in their offspring (persisted for at least 90 days). However, vaccination with the single low dose of vaccine only brought about 42 days of MDAs persistence in their offspring. The effect of MDAs on active immunization with SVA vaccine in offspring was further evaluated, which showed that vaccination of the SVA vaccine in the presence of MDAs at the titer of ≈1:64 or less could overcome the MDAs’ interference and give rise to effective antibody response. This will help for establishing the optimal times and schedules for SVA vaccination in pigs.

## 1. Introduction

Senecavirus A (SVA), also known as Seneca valley virus, belongs the genus of *Senecavirus*, family *Picornaviridae*. As the only member of genus of *Senecavirus*, although SVA contains a typical picornavirus L-4-3-4 genome layout, its viral genes differ remarkably from those of all other picornaviruses [1,2]. SVA genome is a positive single-strand RNA of approximately 7.3 kb in length; it is composed of a 5′-untranslated region (UTR), a single open reading frame (ORF), a 3′-UTR, and a poly-A tail. Similar to other picornaviruses, SVA encodes a large polyprotein from the single ORF, which is subsequently processed into 12 mature proteins, including four structural proteins VP4, VP2, VP3, and VP1, as well as eight nonstructural proteins L^pro^, 2A, 2B, 2C, 3A, 3B, 3C^pro^, and 3D^pol^ [1].

SVA infection causes typical porcine idiopathic vesicular disease manifested by ruptured vesicles and erosions in the oral cavity, vesicle lesions on snouts and coronary bands, as well as lameness [3], which are indistinguishable with the clinical signs of other vesicular diseases such as foot and mouth disease (FMD) and swine vesicular disease (SVD). SVA, as a newly porcine virus, was originally isolated as a contaminant in the cell culture medium during cultivation of PER.C6 cells in 2002 [2]. The SVA positive cases in pigs was first reported in 2007 in Manitoba, Canada [4], and it was supposed to be an etiologic agent of vesicular disease in 2010 in Indiana, US [5]. It is speculated that the virus may have been circulated in pigs for years earlier than when it was first defined as an etiologic agent of swine vesicular disease. Although swine is currently considered as a natural host of SVA, the specific SVA antibodies in cattle and mice have been detected. In addition, SVA has been found and isolated from mouse feces, mouse small intestine, and even environmental samples [2,6]. Exposure to SVA does not give rise to infections in humans [7,8]. SVA does not replicate in normal human cells [8], whereas it can propagate in human tumor cells [9,10]. Whether SVA is a potential health risk for other animals remains unknown.

SVA infection in pigs only sporadically occurred in the US and Canada before 2014 [11,12]. However, since the end of 2014, continuous outbreaks of SVA infection in pigs were reported in different geographical regions in Brazil and then quickly reported in the US, China, Colombia, Thailand, as well as Vietnam with an expanded geographical distribution [3,6,13,14,15,16,17,18]. Moreover, the recombination among SVA strains has been reported recent years [19], suggesting a continuous evolution of SVA. To limit the spread of SVA, a series of diagnostic methods have been established and used for surveillance of SVA in pigs [20,21,22,23,24], and our lab has developed an inactivated vaccine previously that can protect pigs against SVA infection [25]. Appropriate immunization schedules are critical for control of diseases. The maternally derived neutralizing antibodies (MDAs) are important for newborn pigs, which might also hinder humoral responses under inappropriate vaccination [26]. To avoid MDAs interference, the duration of the persistence of MDAs transferred from sows to piglets has to be monitored, and it will assist planners to develop an appropriate immunization schedule. Up to now, the effect of SVA-specific maternal antibody interference to the adaptive immunization in piglets has not been assessed. The principal aim of this study was to evaluate the MDAs (SVA-specific) interference in the piglets and timing of administration of the first dose of SVA vaccine.

## 2. Materials and Methods

### 2.1. Ethics Statement

All the animal experiments described in this study were approved and implemented according to the requirements and management guidelines of the Gansu Animal Experiments Inspectorate and the Gansu Ethical Review Committee (License no. SYXK (GAN) 2017-003).

### 2.2. Study Design

**Figure 1:**
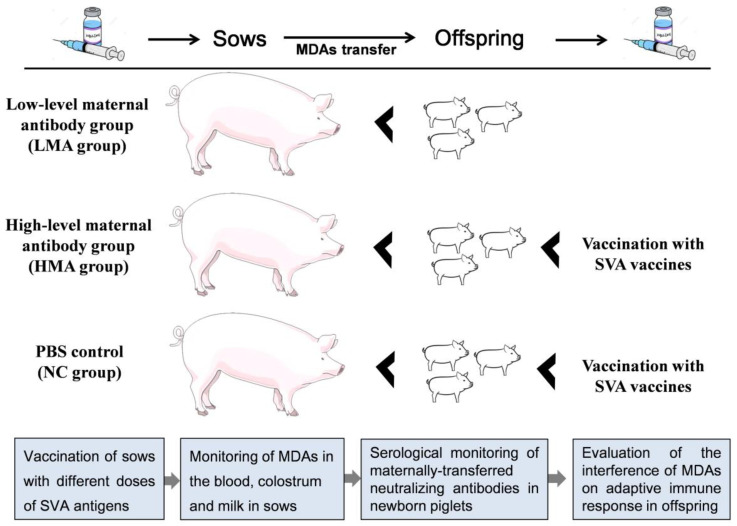
Schematic representation of the experimental design in this study.

The schematic diagram of the animal experimental design is shown in Figure 1. Nine sows (SVA-seronegative) were divided into three treatment groups, as shown in the schematic diagram: (1) the PBS control group, (2) low-level maternal antibody group (LMA group), and (3) high-level maternal antibody group (HMA group). The sows in the PBS control group received 4 mL of adjuvant solutions in sterile PBS on the day 38 of pregnancy. The sows in LMA group received 4 mL of vaccine containing 0.5 μg of SVA antigen on the day 38 of pregnancy. The sows in HMA group received 4 mL of vaccine containing 4 μg of SVA antigen at 38 days of pregnancy and got a booster immunization on day 78 of pregnancy (4 μg of antigen). All the pigs received equal volumes of injections during the vaccination. The neutralizing antibodies in the blood and colostrum/milk in the sows were determined by virus neutralization test (VNT). As for the piglets, the blood was collected after birth at different intervals. In the HMA group, three litters of piglets were vaccinated intra-muscularly in the neck with SVA vaccines at 30, 90, or 120 days of age. Three litters of piglets from the PBS control group were also vaccinated with SVA vaccines at 30, 90, or 120 days of age, which was used as a control for the HMA group. The MDAs’ transfer and its effect on antibody dynamics in offspring was evaluated.

### 2.3. Preparation of SVA Inactivated Vaccines

BHK-21 cells were used to multiply SVA (CH-FJ-2017 strain). The clarified SVA cultures were inactivated using the aziridine compound binary ethylenimine (BEI) to prepare viral antigens as described previously [25]. The inactivated virus cultures were confirmed for loss of infectivity in BHK-21 cells. To prepare the formulated vaccine, the inactivated antigens were purified by sucrose density gradient centrifugation. Montanide ISA 206 (Seppic), an oil adjuvant, was utilized as the adjuvant to enhance the antigen-induced antibody responses. The water-in-oil (W/O) SVA vaccines were prepared by properly mixing the BEI-inactivated SVA antigens and the Montanide ISA 206 adjuvant to a stable emulsion as previously described [27].

### 2.4. Animals, Immunization Experiments, and Sample Collection

Ten days before mating, 9 healthy sows (SVA-seronegative) with similar age and body weight were divided randomly into three groups: (1) the PBS control group (NC #1, NC #2, and NC #3), (2) the LMA group (no. #1, no. #2, and no. #3), and (3) the HMA group (no. #4, no. #5, and no. #6). As for the immunization method, all the pregnant sows were vaccinated intra-muscularly in the neck with different doses of SVA vaccines as described in the “Study design” section. Blood was collected via jugular venipuncture from the sows on the day 15 before farrowing and on the day of birth, respectively. The umbilical cord blood was also obtained on the day of birth. The neutralizing antibodies in the blood were detected by the VNT. The colostrum and the milk from the sows were collected on day 0, 1, 2, 3, 6, 7, and 14 after farrowing. The neutralizing antibodies in the colostrum/milk were determined by VNT. The MDAs levels in all of the piglets right after intake of colostrum was measured and monitored before getting vaccinated. The offspring of the sow NC #1 (in the PBS control group) and no. #4 (in the HMA group) were immunized with SVA vaccines (2 μg of antigen) at 30 days of age. The offspring of the sow NC #2 and no. #5 were immunized with SVA vaccines (2 μg of antigen) at 90 days of age. The offspring of sow NC #3 and no. #6 were immunized with SVA vaccines (2 μg of antigen) at 120 days of age. The antibody responses in the vaccinated piglets were evaluated by VNT after vaccination.

### 2.5. Immune Response Measurement by Virus Neutralization Test

The SVA-specific neutralizing antibody titers in the milk and serum of the sows and their offspring were measured by virus neutralizing antibody test (VNT). The VNT was carried out using the method described previously [25,28,29]. Briefly, two-fold serial dilutions of the prepared samples were performed by transferring equal volumes from one to another. Then, 50 μL of diluent was added to each well of the 96-well tissue culture plate. The 50 μL of prepared SVA (200 TCID_50_) was added to all wells except for the control wells. Then, the mixtures were incubated at 37 °C for 1 h in a CO_2_ incubator. Then, 50 μL of diluted BHK-21 cells (10^6^ cells/mL) in MEM medium supplemented with 8% FBS were added to each well. Endpoint titers were calculated at 72 h post-infection (hpi) and expressed as the reciprocal of the final dilution that led to the neutralization of the virus activity by 50%.

### 2.6. Infection of Pigs

The vaccinated pigs were infected with 3 mL of SVA (10^9^TCID_50_/mL) by intranasal routes as previously described [25]. All the challenged pigs were monitored daily for clinical signs, and the blood samples were collected daily after the virus challenge [30]. The viral RNA copy in the blood was detected by the quantitative real-time PCR method.

### 2.7. Quantitative Real-Time PCR

Total RNA was extracted using the RNeasy kit (Qiagen, Hilden, Germany) following the protocol provided by the manufacturer. The cDNAs were synthesized using 2 μg of the extracted RNAs as templates. The reverse transcription reactions were performed using random hex-amer primers and M-MLV reverse transcriptase (Life Technologies, Carlsbad, CA, USA). Then, the cDNAs were detected using the quantitative PCR method in a Mx3005P qPCR System (Applied Biosystems) as described previously [21]. The CT value greater than 35 was determined to be negative.

## 3. Results

### 3.1. Antibody Responses in Sows after Vaccination with SVA Vaccine

**Figure 2:**
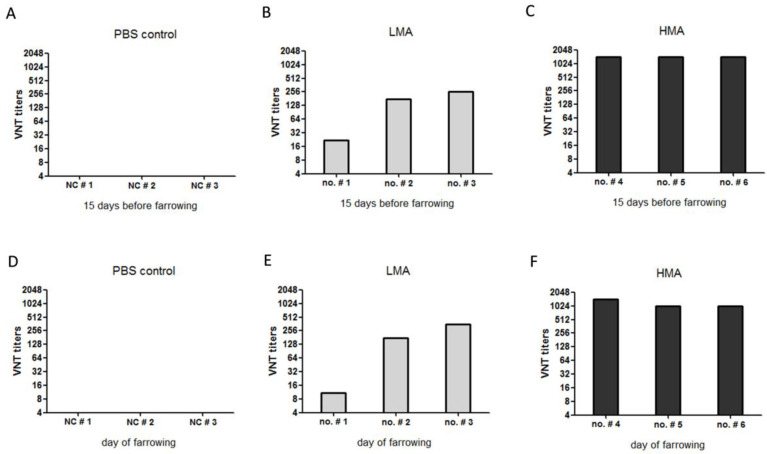
Detection of anti-SVA antibodies in sows after vaccination with SVA vaccines. The serum samples of the sows in the PBS control group, the LMA group, and the HMA group were collected 15 days before farrowing (**A**–**C**) and on the day of farrowing (**D**–**F**) respectively; the antibody titers were assessed by VNT analysis.

The serum samples collected from all the sows of three groups (the PBS control, LMM group, and HMA group) at 15 days before farrowing were subjected to VNT analysis. The vaccination-triggered antibody responses were observed in all of the sows in both the LMA group and HMA group. The sows in PBS control group showed no SVA-specific neutralizing antibody responses (Figure 2A). The sows in the LMA group reflected neutralizing antibody titers of 1:22 to 1:256 (Figure 2B), with sow no. # 1 showing the weakest antibody response (1:22). All the sows in the HMA group developed strong neutralizing antibody responses with the neutralizing antibody titers greater than 1:1024 (Figure 2C). The neutralizing antibody titers in the serum of all the sows on the day of farrowing were also measured. No neutralizing antibodies were detected in the PBS control sows (Figure 2D). The sows in the LMA group showed neutralizing antibody titers of 1:11 to 1:360. As expected, sow no. # 1 still showed the weakest antibody response (1:11) (Figure 2E). The sows in the HMA group had considerably higher neutralizing antibody titers of 1:1024 to >1:1024 (Figure 2F). Upon the designed vaccination with SVA antigens, we acquired the sows containing different levels of SVA specific neutralizing antibodies.

### 3.2. Neutralizing Antibodies in Colostrum and Milk in Sows

**Figure 3:**
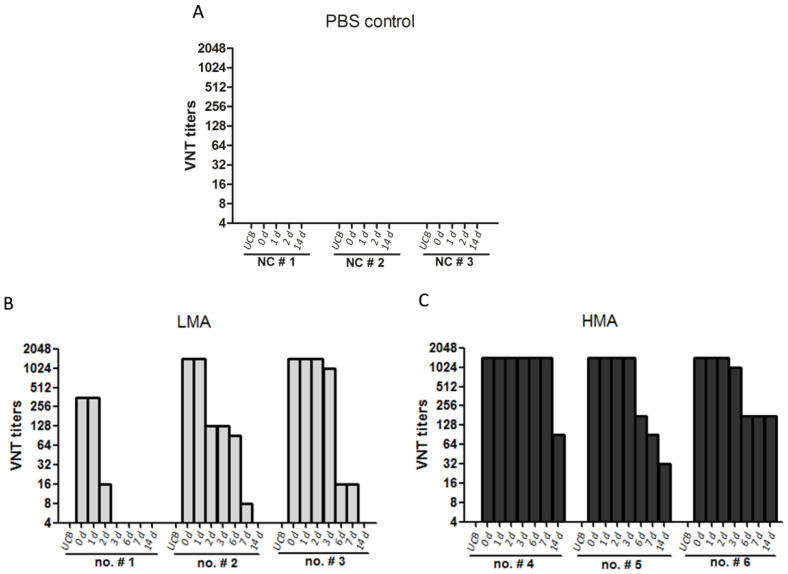
Dynamics of the MDAs in the colostrum/milk of sows immunized with SVA vaccines. The umbilical cord blood (UCB), the colostrum (day 0), as well as the milk secreted on day 1, 2, 3, 6, 7, and 14 after farrowing were collected from all the sows, and the SVA-specific neutralizing antibody titers at the indicated days were evaluated by VNT analysis, respectively. PBS group (**A**), LMA group (**B**), and HMA group (**C**).

To monitor the dynamics of MDAs in milk (also including the colostrum) of the sows throughout lactation, the colostrum (day 0) and milk on day 1, 2, 3, 6, 7, and 14 after farrowing were collected respectively, and the neutralizing antibody titers were evaluated by VNT. No specific SVA antibodies were detected in the colostrum and milk from the sows in the PBS control group (Figure 3A). In the LMA group, the antibody is concentrated in the milk gland (also containing more secretory antibodies) from the serum of the sows. Therefore, the neutralizing antibody titers were extremely higher in the colostrum (1:360 to >1:1024) (Figure 3B) than that in the serum (1:11 to 1:360) (Figure 2E) collected on the day of farrowing. However, the antibody titers in the milk began to wane quickly from the second or third days after farrowing. The antibody level in the milk from sow no. #1 (showing the weakest antibody response after vaccination) dramatically decreased on day 2 and vanished on day 3 after farrowing. For the other two sows, the antibody titers quickly decreased on day 2 or 3 and disappeared on day 14 after farrowing (Figure 3B). In the HMA group, the neutralizing antibody titers in all the colostrum samples were greater than 1:1024 (Figure 3C), and the titers were maintained in higher levels until the 3rd day after farrowing; then, they steadily decreased over time, with two sows (no. #4 and no. #6) still revealing higher titers of 1:90 to 1:180 on day 14. This suggested that the vaccination of sows with a high dose of SVA vaccines followed with a booster immunization resulted in a longer duration of MDAs in the milk. Additionally, the antibody titers in the umbilical cord blood from all of the sows were measured. Pigs do not receive MDAs through umbilical cord blood. As expected, there were no SVA-specific antibodies present in the umbilical cord blood, suggesting that SVA MDAs were transferred to offspring through the colostrum/milk but not umbilical cord blood (Figure 3).

### 3.3. Transfer of Maternal Neutralizing Antibodies from Sows to Piglets

**Figure 4:**
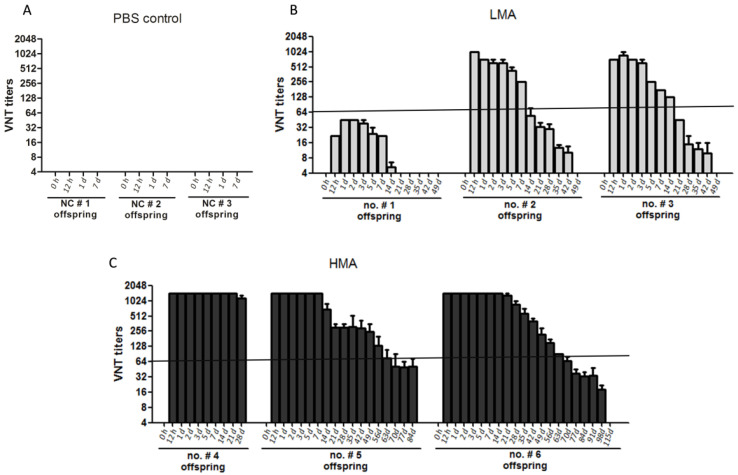
Dynamics of the MDAs transferred from sows to the offspring. The serum samples of the piglets in the PBS control group (**A**), the LMA group (**B**), and the HMA group (**C**) at the indicated time-point during intake of colostrum/milk were collected and subjected to VNT analysis, respectively.

Neutralizing antibody levels in milk from sows were strongly associated with the antibody titers in their offspring. Without MDAs, the piglet will be highly susceptible to infection. The piglets were weaned at 28 days of age. The antibody titers in the offspring (serum samples) of all the sows used in the experiments were assessed. No SVA-specific antibody was detected in the serum of piglets from the PBS control group (Figure 4A). The SVA-specific antibodies were absent in all the newborn piglets from the LMA and HMA groups before the first suckle (0 h), and the antibodies could be detected after intake of colostrum (Figure 4B,C). The MDAs levels in the serum of piglets from sow no. #1 in the LMA group were extremely low (1:16 to 1:45), with a duration of ≈7 days and then faded away. The MDAs were almost vanished on day 14 even still feeding the milk to the piglets. As for the offspring of the other two sows (no. #2 and no. #3) in the LMA group, the MDAs titers started to decline from 5 to 7 days after birth. The antibody titers in most of the piglets were about 1:64 on the 14 days after farrowing and then gradually decreased to about 1:8 to 1:32 at the weaning day (day 28 after birth), and it completely vanished 21 days after weaning (day 49 after farrowing) (Figure 4B). The antibodies in the serum of the piglets from the sows of the HMA group were extremely high before weaning, with the neutralizing antibody titers of 1:256 to >1:1024 (Figure 4C). Then, the titers faded away slowly, decreasing to about 1:64 after ≈60 days and disappearing ≈115 days after birth (by monitoring the offspring of sow no. #6). Therefore, as for the piglets, the HMA group showed much longer MDAs maintenance than the LMA group.

### 3.4. High Titers of MDAs Interfere with SVA Antigen Vaccination of the Offspring

**Figure 5:**
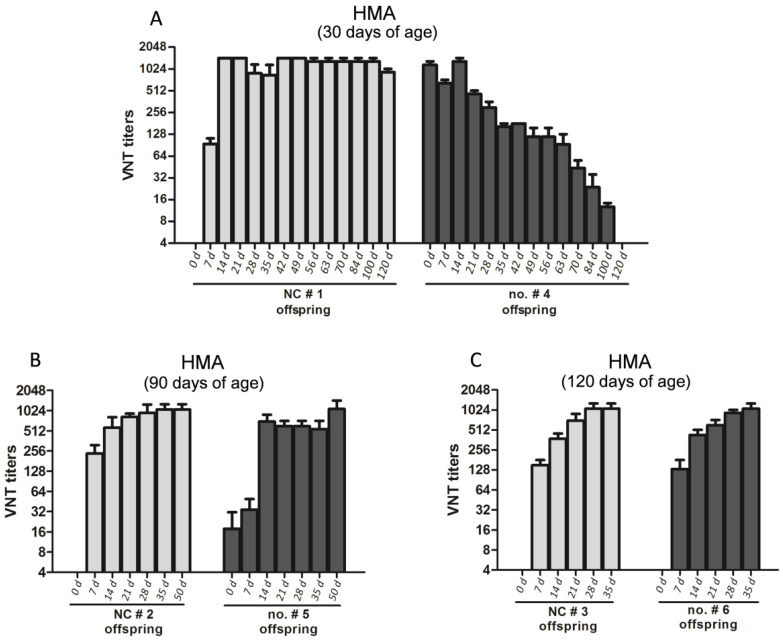
Evaluation of the effect of MDAs on adaptive immune response within the offspring. The offspring of the sows in the PBS control group and the HMA group were immunized with inactivated SVA vaccine (2 μg of antigen) at 30 (**A**), 90 (**B**), or 120 (**C**) days of age, respectively. The serum samples were collected at the indicated time-point after vaccination and subjected to VNT analysis to evaluate the interfere of the MDAs. All measured values were expressed as the means with standard deviation.

To evaluate the interference of the MDAs to the adaptive immune responses in the piglets, three litters of pigs in the HMA group were immunized with SVA vaccines at 30, 90, or 120 days of age. The offspring from sow no. #4 was vaccinated at 30 days of age. The offspring from sow no. #5 was vaccinated at 60 days of age, and the offspring from sow no. #6 was vaccinated at 120 days of age. The neutralizing antibody titers were measured at different time-points post vaccination. The three litters of pigs in the PBS control group (SVA-seronegative) were subjected to the same treatment and used as the controls for the HMA group. Comparing to the piglets from the control group, the MDAs apparently caused interference to piglet humoral response development after immunization with SVA vaccine at 30 days of age (Figure 5A). The control piglets revealed strong antibody response after immunization with SVA vaccine at 30 days of age and showed a long-term maintenance of high titers of neutralizing antibody (sustaining the titers of >1:1024 even at the 120 days after vaccination). However, in the offspring from sow no. #4, the titers began to decline on day 14 after immunization and completely vanished 120 days after vaccination (Figure 5A). In the course of monitoring MDAs titers within the offspring from sows no. #5 and no. #6, we determined that the titers were ≈1:22 to 1:64 at 90 days of age (Figure 4C). Therefore, we vaccinated the offspring of sow no. #5 with SVA vaccines at 90 days of age. As expected, no significant intervention caused by the MDAs was observed. The titers in these pigs vaccinated at 90 days of age displayed a similar antibody response trajectory over time as that in the PBS control group (Figure 5B). As for the offspring from sow no. #6, which were immunized by the SVA vaccines at 120 days of age, no MDAs interference to the adaptive immunization was observed as well (Figure 5C). These data suggested that a high level of SVA MDAs interferes with the adaptive immunization in piglets, and vaccination of SVA vaccine when the MDAs titers were less than 1:64 (at ≈60–90 days of age) could overcome the interference of MDAs.

**Figure 6:**
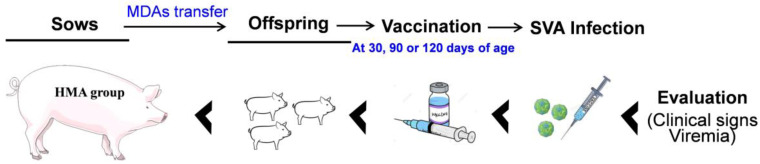
Schematic representation of infection of the offspring in the HMA by SVA.

The viral infection assay was also carried out in the vaccinated offspring (Figure 6). The offspring of sow no. #4 were infected with SVA by intranasal routes on day 125 after vaccination (155 days of age), the offspring of sow no. #5 were infected with SVA on day 65 after vaccination (155 days of age), and the offspring of sow no. #6 were infected with SVA on day 35 after vaccination (155 days of age). The clinical signs were observed, and the viremia were detected in the offspring of sow no. #4. The fluid-filled vesicles on the snout and ulcerative lesions on the coronary band were observed in the offspring of sow no. #4, and the viral RNAemia could be detected by qPCR (targeting the viral polymerase gene 3D). However, the offspring of sows no. #5 and no. #6 showed no clinical signs (no lesions or clinical diseases) and no viremia (no CT value) (Table 1). This indicated that high levels of SVA-specific neutralizing antibodies could provide adequate protection against SVA infection.

## 4. Discussion

The outbreaks of SVA infection in pigs have been increasing in many countries and regions in recent years, which has become a substantial problem for the pig industry [31]. SVA infection causes typical clinical manifestation of vesicular disease, which is indistinguishable with FMD. In addition, SVA infection was associated with epidemic transient neonatal mortality [32,33]. The phylogenetic analysis indicates that SVA is evidently set apart from foot-and-mouth disease virus and other picornaviruses and therefore has been classified into a newly created genus. Our previous study also suggested that there was no cross-protective efficacy of foot-and-mouth disease virus vaccine against SVA infection [16]. Vaccination is a most effective method for the prevention and control of various infectious diseases [34]. We have developed a potential SVA vaccine candidate previously [25], and it is now under a clinical trial in more pigs. The neutralizing antibody dynamics in sows and their offspring after SVA vaccination were evaluated in this study.

Maternal antibody interference is independent of the type of vaccine being used. A cross-link between B cell receptor (BCR) with the Fcγ-receptor IIB by a vaccine–antibody complex results in the B cell inhibition [35,36]. Therefore, maternal antibodies inhibit the generation of antibodies by inhibition of B cell responses through epitope masking. In addition, the binding of maternal antibody to the Fcγ-receptor IIB of B cell also contributes to B cell inhibition [36]. Maternal antibodies are very effective in protecting newborn animals against most infectious diseases, and the maternal antibody titer is of critical importance to newborn pigs. Without maternal antibodies, the piglet is highly susceptible to infection. The MDAs transferred by sows to their offspring through colostrum and milk play important roles in preventing pathogen infections before getting vaccinated. In addition, the growth-suppressive effects caused by novel antigen immunization in offspring have been reported [37,38], and the presence of MDAs ameliorates the growth-suppressive effects caused by vaccination. Therefore, it is suggested that MDAs cut down costs of an immune response [39]. In the present study, the sows were immunized during gestation with inactivated SVA vaccines through the intra-muscular route. We found that MDAs induced by vaccination were efficiently transferred to the offspring by feeding colostrum and milk. Particularly in the HMA group, the MDAs of SVA were maintained up to ≈98 days after birth (Figure 4C). However, if insufficient immunization is performed in sows (see LMA group), the MDAs would disappeared quickly (maintaining for one to six weeks after birth) (Figure 4B). This indicated that the effective vaccination of sows using SVA vaccines will contribute to a long-term duration of the persistence of MDAs in their offspring and therefore may benefit the piglet growth.

We also confirmed that the SVA antigen immunization-induced specific neutralizing antibodies were only transferred from sows to the offspring through drinking colostrum and milk but not through the umbilical cord blood on the day of farrowing (Figure 3). Therefore, appropriate and timely feeding of colostrum and milk are critical for providing plenty of MDAs to the piglets. Booster immunization of vaccine in sows increases the MDAs in the colostrum and milk, which is vital for supplying better maternal protection in the offspring. SVA has been detected and isolated from piglets, and high rates of pig neonatal mortality have been reported in many SVA infection cases [31,32,40]. If an outbreak of SVA infection occurs, a well-planned immunization of sows will potentially provide efficient maternal protection required to prevent neonatal disease or death provoked by SVA infection. Vaccination of sows using a high dose of SVA vaccines followed by a booster immunization could provide about 3 months duration of persistence of MDAs in the offspring, which can provide a better maternal protection.

**Figure 7:**
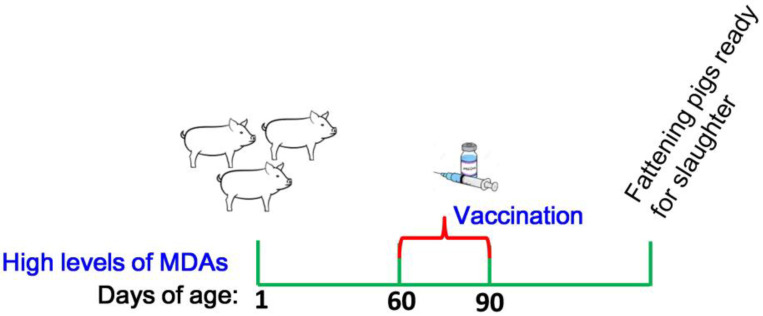
Recommended SVA vaccination schedule for piglets with high levels of MDAs. We recommended that the piglets with high levels of MDAs receive the vaccination of SVA vaccines on days 60 to 90 after farrowing.

Although the MDAs are crucial for offspring to prevent early viral infections, when the antibody titer declines to a very low level, it will not provide complete protection against viral infections. Hence, the adaptive immunization is essential for strengthening antibody response in offspring. However, the interference by high titers of MDAs prevents the development of adaptive immune response [36]. The presence of MDAs should be taken into consideration when carrying out the vaccination. The kinetics of MDAs in the offspring has to be determined to confirm that the vaccination is performed in the right condition and at the right time. The aim of monitoring the SVA antibodies in the offspring in this study is the timing of administration of the first dose of SVA vaccine and reducing interference by MDAs. For this purpose, the effect of MDAs on adaptive response in the offspring was evaluated. We found that if the sows were vaccinated with a high dose of vaccines followed by a booster immunization, the MDAs in the piglets at 30 days of age (antibody titer is ≈1:256 to 1:1024) will interfere with the efficacy of the first vaccination. Immunization of SVA vaccine at 90 days of age (antibody titer is ≈1:22 to 1:64) after birth could overcome the maternal antibody interference and give rise to effective adaptive immune response. This will provide reference data for generating optimal vaccination schedule for piglets with high levels of MDAs. We recommended that the offspring with high levels of MDAs should receive the vaccination of SVA vaccines on days 60 to 90 after farrowing (Figure 7).

## 5. Conclusions

In conclusion, our study has determined the kinetics of MDAs in the colostrum/milk from sows throughout lactation and the persistence trajectory of transferred antibodies in the offspring. Meanwhile, we evaluated the interference of MDAs on adaptive immune response in the offspring and identified the optimal time point for getting vaccination to overcome the interference. This will help establish appropriate immunization schedules for using SVA vaccine in pigs.

## Figures and Tables

**Table 1:** The clinical signs and viremia state in the offspring infected by SVA.

Offspring	Clinical Signs (Fluid-Filled Vesicles and Ulcerative Lesions)	Viremia (Cut-Off CT Value Is 35)
No. #4	+	+
No. #5	−	−
No. #6	−	−

Notes: “+” represents positive, and “−”indicates negative.
